# Pleurisy in Children

**Published:** 1883-01

**Authors:** Wm. T. Plant

**Affiliations:** Prof. Dis. of Children in Syracuse University


### PLEURISY IN CHILDREN.

*Gentlemen:* Our subject for to-day is pleuritis. This is not, as you are aware, a disease of early life exclusively; it has even been asserted to be extremely rare at this period. There is good reason to believe, however, that while the disease is rather frequent, its recognition is somewhat rare.

I wish mainly to set before you the peculiarities that characterize pleurisy in infancy and childhood, so that you may be left without excuse if you fail of that diagnostic precision in which so many before you have been found wanting.

In the young as in the mature, pleuritis is almost always unilateral, and that is a blessing, for thereby we are furnished with a standard of comparison.

It is practically important that you should know that it occurs under different conditions. It may be *primary*—standing apart from any other disease; or it may be *secondary*—that is, attendant on and sequent to some other malady, as pneumonitis, scarlet fever, nephritis, rheumatism or pulmonary consumption.

As a primary affection its usual cause is taking cold. It may happen to the youngest infant, though it is mostly met with in children who are older and more liable to exposure. In grown people the initial symptom is a chill. Not so in infancy, and not generally so in childhood. Sometimes vomiting is the first thing noticed; sometimes a convulsion or a series of them. But usually the first symptom of prominence is pain—a stitchy, stinging pain. Though infants cannot tell you this, the fact of pain is often made evident by fits of crying and screaming and a disinclination to be moved from a chosen position. Older children will indicate the seat of pain. In most cases, perhaps, it is in one side near the nipple; but quite often it is not in the thorax at all, but in the upper part of the abdomen, and the child's constant wail may be that his "belly hurts." I would have you make a mental note of this, for not a few children have been treated for colic when the real trouble was pleuritic. I suppose the reason of this is to be found in the fact that the lower intercostal nerves are distributed to the integument of the abdomen. The pain of pleuritis in early life varies greatly as to intensity. Sometimes the little one appears to be in the extremest distress, and there may be such tenderness of the affected part that the least pressure causes flinching. In other cases the pain is moderate and not lasting. Though I cannot give you the reason, I may mention the fact that the pain may remain limited to one small spot, though all the pleura of that side may have become inflamed.

I may as well tell you here that you will sometimes fall in with cases of pleurisy that are latent as to pain and other prominent symptoms. A child that had not been known to be seriously ill is brought to you for an opinion as to the cause of its failing health. You examine it and find one side of the thorax full of fluid. Insidious pleurisy is rather frequent in early life, especially in connection with scarlet fever and some other diseases.

The next symptom that will in most cases engage your notice is the cough. A child in the first days of pleuritis handles its cough with the greatest caution. It is short, dry and frequent, and the pain that it causes and the efforts to suppress it are often depicted in the features. But the cough is as variable as the pain. In some cases it is well nigh constant; in a few so slight as scarcely to attract notice. But please to notice that the cough *follows* the pain—the latter generally having a lead of half a day or more.

Another point is the fever. Pleuritis, like other affections ending in *itis*, is attended by a rise of temperature. I think it is seldom quite as high as in acute pneumonia. The difference in surface heat between these two divisions may be strikingly evident to the hand. In pneumonitis the integument is often "burning hot;" in pleurisy it feels but little warmer than nature. In pneumonitis also the face is flushed, often crimson; in pleuritis if there is a little flushing at first it soon subsides and leaves the countenance pale and often rather sallow. Notice also the decline of temperature in the two diseases. In acute pneumonia it is sudden; at the end of a week or thereabouts the crisis occurs and the temperature falls quickly—in one day—to the normal or even below it. But in pleuritis the decline is always gradual. Often two or three or more weeks pass before it drops to the standard of health. The pulse is, of course, quickened in its pace, and there are the usual attendants of the febrile state.

Occasionally, in the first days, when the fever is at the highest, there is severe headache and active delirum ; and if there is also vomiting and constipation you may lean towards a diagnosis of cerebral inflammation. But consider and weigh all the symptoms and carefully examine the chest and you will seldom go wrong.

Another feature of this disease that claims your attention is the breathing. It is hurried, but less so as a rule than in pneumonia. If you observe it carefully you will be struck with its superficial character. The child prefers to breathe frequently rather than deeply, for it has learned that a full breath excites the cough and causes pain. There is seldom either much dyspnœa or lividity. If the child needs more air, it breathes oftener rather than deeper. Sometimes there is a little expansion of the nares and an expiratory moan, but these features are seldom as prominent as in pneumonitis.

Altogether the child will probably seem to be less ill than are children with acute inflammation of the substance of the lung, nor is there at the end of a few days that sharp turn for the better that characterizes the latter disease. The natural result of an inflammation of the pleura is, as you well know, an increase in its functional activity ; hence an exudation of fibrinous lymph or of serum, or both. Layers of fibrin are deposited on the pleural surfaces while detached shreds and floculi of it float in the fluid that is accumulating within the cavity. In most instances, this fluid is a clear serum ; but here is a point that I would emphasize : in children this fluid has a remarkable tendency to become purulent ; sometimes, indeed, it has this character from the very first. This is empyæma.

The amount of effusion is variable. There may be but two or three ounces, not enough to hamper the lung in its movements ; or there may be sufficient to fill the cavity full and over-full so that the lung, retiring before it, is crowded into a corner at the upper and inner part of the chest—an airless, bloodless, leathery lump.

I hardly need to tell you that as a result of excessive effusion, the diaphragm may be pressed downward, the heart crowded to one side, the intercostal spaces rounded outward and the side considerably increased in its measurement. The increase in size, however, may be difficult to estimate, because the other side may be enlarged as much from the increased volume of the sound lung that now has double work to do.

I have gone somewhat minutely into the general symptoms because the physical signs on which in the pleurisies of adults we can plant ourselves with so much assurance are often in children unreliable and misleading. Especially is this so at first. Auscultation is unsatisfactory, because the child breathes as superficially as possible and the friction sound is seldom caught in infants and young children.

After some days, when considerable effusion has occurred, a diagnosis is not difficult. The flat, toneless thud, and the sense of great resistance on percussion, are of themselves almost conclusive of a fluid accumulation. Above the level of the liquid the sound will be clear and tympanitic. In some instances the diagnosis may be happily confirmed by observing that the upper line of dullness varies with changes in the posture of the child. But often the fluid is confined by fibrinous partitions, or the pleural sac is full, and then this test is not available.

In the adult when the effusion is large, all respiratory sounds may be bsent and the results of auscultation are only negative ; but in children there is seldom so much fluid between the lung and the chest-wall as to do away with bronchial breathing, and quite often the vesicular murmur may still be faintly heard. This will not be so, of course, when the accumulation is so great and the pressure so long continued as to wholly close the lung to the entrance of air. But in any event the contrast between the diminished air sounds of the crippled side, and the exaggerated respiration and hyper-resonance of the sound side will be so pronounced that there should be no error of diagnosis. When there is much effusion it may be both seen and felt that the usual mobility of that side is lessened.

Some writers speak of a change of shape as a sign of large effusion, consisting in lateral flattening and and anterior bulging.

You will not forget that in the young pleurisy and pneumonia are often concurrent—pleuro-pneumonia. In that case you will recognize the prominent symptoms of both diseases and you will give a guarded prognosis, for the condition is one of extreme peril.

In early, as in mature life, pleurisy may terminate in different ways. In many—in most cases—the fever ceases within a few days; the exudation is speedily absorbed; the lung regains its former volume, and within two or three or four weeks the child may be as well as ever. In some cases, and especially if the pleurisy is secondary to other disorders, the child may die at length from disease and exhaustion. In many instances, the fluid, if not purulent at first, soon becomes so. I think I have already stated that suppurative pleurisies are much more frequent in the young than in older people. Secondary pleuritis is very often of this character. Empyæma is always a serious disease. It is true that when the quantity of pus is small it may be disposed of through fatty degeneration and absorption, but not so, I think, if the cavity is full or nearly full of pus. It is retained; and before long there are symptoms of pyæmic infection, such as high fever, exhausting night sweats and rapid wasting. After some weeks or months, if the child lives so long, unless our art has provided an outlet for the pus, nature attempts its evacuation either through a spontaneous opening in the chest wall, or internally through the air tubes or esophagus, or possibly downward through the diaphragm. In children, evacuation through the outer wall seems to be nature's favorite method.

But even when the drain has been established the child does not always recover. The production of pus may keep pace with its discharge until the patient sinks from exhaustion or falls into a hasty consumption. If it lives and the discharge at length ceases, there is apt to be retraction of the side and spinal curvature resulting from atmospheric pressure. In children, however, much oftener than in adults, the crippled lung may by slow degrees become re-inflated and reach at length its former volume, and in this way a very considerable deformity may in time be overcome.

"Prompt and very efficient blood-letting is indispensable in the treatment of this form of pectoral inflammation. Blood should be freely drawn with the lancet until a decided impression is made on the pulse. The early application of leeches to the chest is also a highly important measure. As soon as the momentum of the circulation has been moderated a blister ought to be laid over the breast. The bowels must in the first place be freely evacuated by an efficient dose of calomel and rhubarb, and kept in a loose state throughout the course of the disease by small doses of calomel and ipecac, or suitable portions of epsom salts."

I have quoted these lines from a great authority in his day,* partly that you may see how tenderly the little ones of thirty or forty years ago were treated, but chiefly to caution you against such counsel. Do nothing of the kind. Do just the other way. Avoid reducing measures and seek to preserve the child's strength.

The truth is that most cases of primary pleurisy tend to speedy recovery without medical treatment. Yet we are not on that account to withhold our ministrations.

If the pain is severe, considerable relief may usually be obtained by hot poultices—linseed is as good as any—so covered as to retain their heat. More rapid and complete relief may be had by the hypodermic use of morphine—one-thirtieth of a grain for a child of one year.† When the pain is referred to the abdomen a broad bandage so applied as to restrain abdominal and diaphragmatic movement may give some relief.

To quiet the cough at night and secure rest Dover's or Tully's powder may be given in doses of from one to three grains, according to the age. At first, while the fever lasts, the diet should be light and simple; later it should be nutritive but plain. Constipation is to be obviated; beyond that I do not believe cathartics are of service.

Some cases require more decided treatment. In weakly children, especially if the pleurisy is secondary, absorption may be for a long time at a stand still. Your first duty in such cases is to determine whether the fluid is serous or purulent. This is easily done by passing the hypodermic needle through an intercostal space in the lower half of the chest. The back is preferable because the child is less ter-

---

* Eberle on Children. † Eustace Smith.

rified when not a witness of the procedure. If clear serum is withdrawn you are justified in resorting to medical means to hasten its absorption. Among these means diuretics have always been in favor. If unable to devise a better, you may use a formula something like this:

| | | |
|---|---|---|
| R | Potassi iodidi.......... | ʒ ii |
| | Potassæ nitratis........ | ʒ i |
| | Infusi digitalis.......... | ʒ ii |
| | Syrupi simplicis........ | ℥ iss |
| | Elix. simplicis.......... | ℥ i |
| | Aquæ ad.............. | ℥ iv. |

Teaspoonful once in four hours for a child of three or four years old.

Tonics do well for these cases, and about the best of them is the old muriated tincture of iron. From five to ten drops with syrup and water will not be too much for a child from one to three years old.

We likewise have local treatment for promoting absorption. Inunctions with blue ointment have, I am glad to say, fallen into merited disuse. The compound iodine ointment is a good remedy. It may be applied over the effusion with suitable friction from one to three times daily. Eustace Smith prefers the liniment of iodine to any other form. He paints a spot the size of the palm of the hand twice daily until the skin becomes irritated, and then works a new field. I believe that small blisters removed from place to place—flying blisters as they are called—are about as efficient agents as we have for exciting the absorbents. But blisters, even small ones, are irritating and tantalizing to the young, and I seldom use them if I can serve my ends by other means. Seek in all ways to put your patient in good general condition. "Proper nutrition and good air," says Vogel, "are the main essentials to rapid absorption."

Some late authors recommend a "dry diet" to starve out the effusion. I doubt whether much is to be gained in this way, for the system will not long remain in good condition if deprived of a proper supply of fluid.

By perseverance in the use of the above means, with now and then, if the little one is not weak, a sharp cathartic or a sweat, most serous pleuritic effusions will, after a time, become wholly absorbed.

But if the quantity is excessive, if the mediastinum is crowded to one side and dyspnœa occur, the fluid should be promptly let out. This may be done by a small trocar and canula, or better, perhaps, by the aspirator. It has often been noticed that the removal of a part of the fluid serves as a stimulus to absorption, so that the residue is taken care of without farther instrumental aid.

If, instead of abating at the usual time, the fever continues or increases; if there are profuse night sweats and a growing debility, and if percussion shows that the fluid is not lessening, it probably is, or is becoming, purulent. If this suspicion is confirmed by an explorative puncture, the sooner you tap the better. It is true that nature may establish a drain and make a tardy and (too often) an incomplete cure, for the proportion of empyæmas in children successfully treated by aspiration is much greater than in adults. If the pus re-accumulates after its withdrawal the operation may be again and again repeated.

It seems, sometimes, as if the whole pleura had become converted into a pus-forming membrane, so rapidly is it produced. For these cases I think the better way is to make a counter opening; to wash out the cavity daily with warm water *slightly* carbolized or iodinized, and to insert a drainage tube. In all cases of empyæma, bear in mind the danger of phthisis. Feed your little patient liberally with milk punch, eggs, meat broth and the best food he can digest, and resort early to such agents as cod-liver oil and quinia—*Wm. T. Plant, M. D., Prof. Dis. of Children in Syracuse University, in Obstetrical Gazette.*

